# Chain Topology Engineering in Amphiphilic Block Copolymers: Crosslinking‐Induced Nanodomain Refinement for Ultrahigh Energy Density under Extreme Conditions

**DOI:** 10.1002/advs.202510046

**Published:** 2025-09-15

**Authors:** Fuxing Zhai, Lixin Xu, Huijian Ye

**Affiliations:** ^1^ College of Materials Science and Engineering Zhejiang University of Technology Hangzhou 310014 China

**Keywords:** amphiphilic block polymer, crosslinking network, cyclic stability, energy density, high‐temperature dielectrics, microphase separation

## Abstract

The growing demand for high‐performance capacitors in extreme environments drives the development of polymer dielectrics with high energy capability and thermal stability. Here, an amphiphilic block copolymer constructed via self‐assembly‐induced nanoscale microphase separation is presented, in which fluorinated polyimide (FPI) and flexible polyetheramine (PEA) segments form thermally entropy‐driven domains. Density‐of‐state model reveals significant electronic heterogeneity of the resultant copolymer, in which FPI exhibits an energy gap of 3.49 eV while PEA demonstrates a wide gap of 7.31 eV, resulting in an interfacial offset of 5.28 eV that inherently restrains the migration of charge carriers. A trifunctional crosslinker with dual electron‐withdrawing groups (─CF_3_/─O─) is introduced to refine the PEA phase domain that decreases to 13.7 nm from an initial long period of 19.2 nm. The crosslinking system achieves record‐high energy densities of 11.07 J cm^−3^ (150 °C/700 MV m^−1^) and 6.45 J cm^−3^ (200 °C/600 MV m^−1^) with an efficiency of >90%, which is attributed to the diminished hopping distance of charge carriers under high‐temperature electric field. The received copolymer film retains 1.56 J cm^−3^ and an efficiency of 94% with fluctuation of <5% after 10^5^ charge–discharge cycles at 200 °C and 300 MV m^−1^. By synergizing amphiphilic energy‐level engineering with entropy‐driven phase refinement, this strategy establishes a robust material platform for a high‐temperature capacitor, balancing ultrahigh energy storage and dielectric reliability.

## Graphical abstract

An amphiphilic block copolymer is constructed via self‐assembly‐induced nanoscale microphase separation. A crosslinker with dual electron‐withdrawing groups (─CF_3_/─O─) is introduced to refine the PEA phase domain. The crosslinking film achieves record‐high energy densities of 11.07 J cm^−3^ (150 °C/700 MV m^−1^) and 6.45 J cm^−3^ (200 °C/600 MV m^−1^) with an efficiency of >90%.

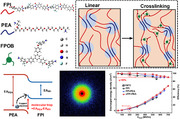

## 1 Introduction

With the rapid development of electric vehicles and underground energy exploration, the stable operation of electronic devices under extreme temperatures (>150 °C) and electric fields (>500 MV m^−1^) becomes increasingly urgent.^[^ ^1^, ^2^, ^3^ ^]^ As a core component for energy storage and conversion, polymer film capacitors have been deemed a potential candidate due to the outstanding power density, fast charge–discharge rate, and dielectric reliability.^[^ ^4^, ^5^, ^6^, ^7^ ^]^ However, traditional dielectric material such as commercial biaxially oriented polypropylene (BOPP) has an upper operating temperature of ≈85 °C, and the operating voltage is essential to be drastically lowered (30–50%) under high temperature, which severely limits the application in scenarios such as near‐engine working temperature of >120 °C.^[^ ^8^, ^9^, ^10^ ^]^ The polymer film with a high glass transition temperature (*T*_g_) still faces the challenges of leakage current and charge–discharge efficiency under the external field.^[^ ^11^, ^12^, ^13^ ^]^ This bottleneck stems from the intensified charge injection, excitation, and migration under the coupling effects of high‐temperature electric field, leading to a significant increase of the conductivity loss with the decline of breakdown strength.^[^ ^14^, ^15^ ^]^ Therefore, the development of an all‐organic dielectric polymer that combines high‐temperature energy storage capability with dielectric reliability has become a primary solution.

In recent years, various strategies have been proposed to enhance the energy storage performance of polymer film, for example, by doping high bandgap inorganic fillers such as boron nitride nanosheets (BNNS) and alumina (Al_2_O_3_) to generate a charge‐blocking layer.^[^ ^16^, ^17^, ^18^, ^19^ ^]^ However, the dielectric diversity at the interface results in the accumulation distribution of local field under the action of the external electric field.^[^ ^20^, ^21^, ^22^, ^23^, ^24^ ^]^ Molecular engineering modifications, such as introducing a high electron affinity molecular semiconductor to capture carriers with deep trapping sites, can suppress the leakage current and improve the charge–discharge efficiency.^[^ ^25^, ^26^, ^27^ ^]^ Although traditional crosslinking networks restrain the thermal movement of molecular chains, it's difficult to combine high energy density with low loss due to the weakening electric displacement caused by the low‐polarity groups of the crosslinker.^[^ ^28^, ^29^ ^]^ These approaches generally encounter the dilemma of “performance‐process‐cost” trade‐offs, and there is an urgent need to develop an all‐organic material system with intrinsically inhibited charge migration and processability.^[^ ^30^ ^]^

The entropy‐driven self‐assembled microphase separation of amphiphilic block copolymer has been accomplished through spontaneous formation of nanoscale regions,^[^ ^31^ ^]^ and the charge potential barrier from the energy level difference of the block segments has not been explored in the high‐temperature dielectrics yet. The thermodynamic incompatibility between the embedded segments delivers the microphase separation to form a periodic nanostructure without the complex external regulation. The energy level difference between the rigid and flexible phases is rationally designed to yield a natural carrier migration barrier at the interphase region, effectively suppressing the charge jump inside the bulk dielectrics. In this study, the self‐assembled phase separation strategy of amphiphilic block copolymers is introduced into the design of high‐temperature energy storage dielectrics. The FPI‐PEA block copolymer has been constructed through molecular engineering, in which a three‐armed crosslink agent 1,3,5‐tris(4‐(2‐trifluoromethyl‐4‐nitrophenoxy)phenyl)benzene (FPOB), containing strong electron‐absorbing groups, refines the phase region. The resultant copolymer achieves an energy storage density (*U*_e_) of 11.07 J cm^−3^ with charge–discharge efficiency (*η*) of 90% at 150 °C and 700 MV m^−1^, and *U*_e_ = 6.45 J cm^−3^ with *η* = 90% at 200 °C is obtained under 600 MV m^−1^ for the current film. The copolymer film with tailored nanophase demonstrates outstanding charge–discharge stability, e.g., *η* > 90% after 10^5^ field on‐off cycles of 300 MV m^−1^ with testing temperature of 200 °C. The nanoscale spatial decoupling of rigid and flexible phases is generated through the entropy‐driven self‐assembly mechanism, in which the energy level barriers of the interface and the tailored crosslinking network are utilized synergistically to inhibit the charge migration under high field. The self‐assembly process is achieved by combining copolymerization and crosslinking one‐step reaction that's preferred for the scaled‐up production. This study extends the design framework of molecular self‐assembly regulation and aggregation to flexible electronics and high‐power pulse device, which promotes the next‐generation energy storage technology to evolve in the direction of high‐temperature reliability.

## 2 Results and Discussion

### 2.1 Molecular Design and Self‐Assembly‐Driven Construction of High‐Temperature‐Resistant Dielectrics

**Figure 1:**
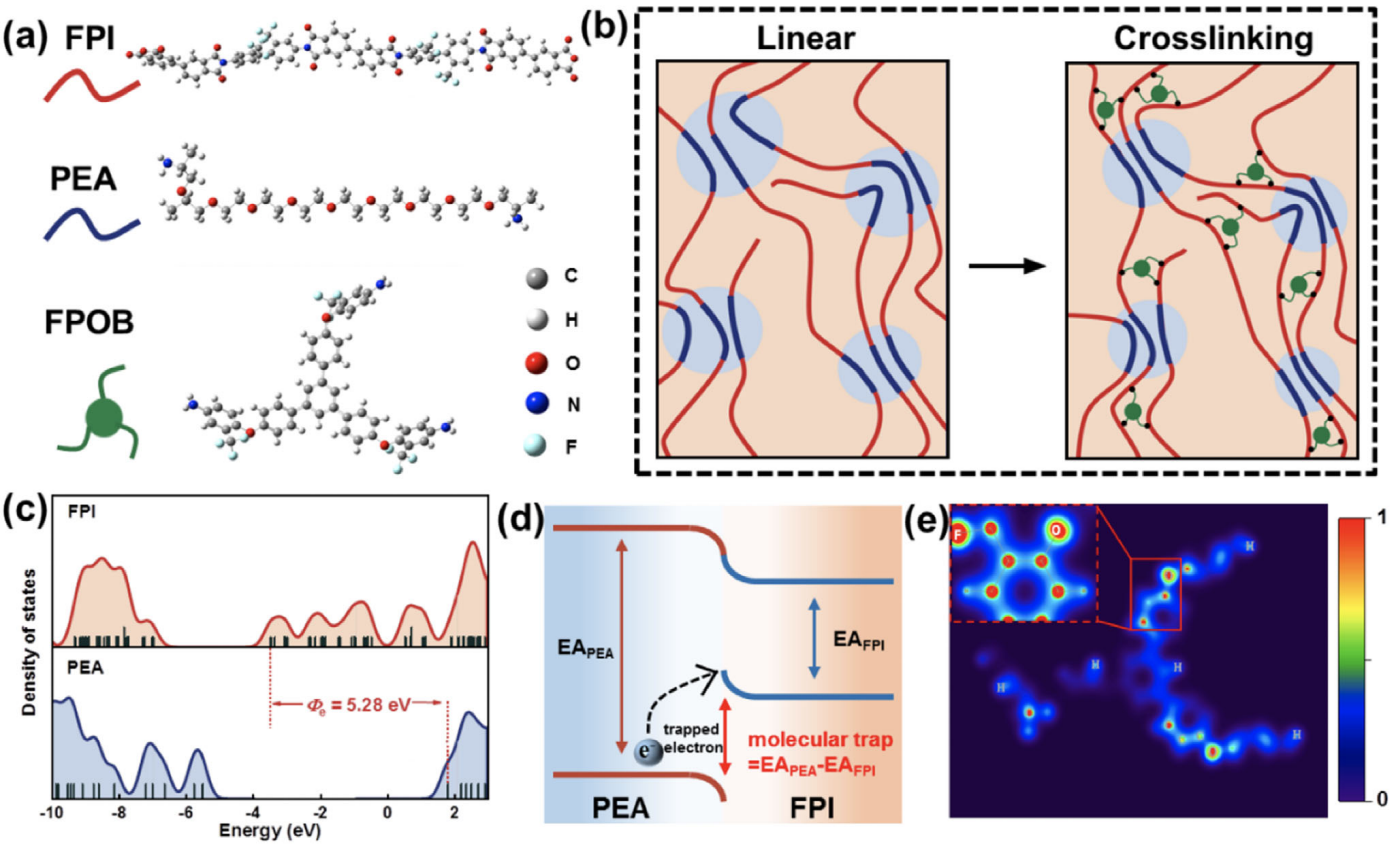
Molecular design and entropy‐driven construction of high‐temperature cFPI‐PEA film: a) geometrically optimized configurations of FPI, PEA, and FPOB, b) schematic structure of microphase separation, c) density of states for FPI and PEA, d) schematic construction of charge traps by difference of electron affinity potentials, and e) contour of electron localization of FPOB molecule.

In the preparation of amphiphilic block polymer, the fluoro‐polyimide (FPI) as rigid segment was composed of 4,4'‐diamino‐2,2'‐bis(trifluoromethyl)biphenyl (TFMB) and 3,3',4,4'‐biphenyltetracarboxylic acid dianhydride (BPDA) monomers, and poly(etherammonia) (PEA) soft segment containing multiple ether‐bonded structure was copolymerized in this study. The crosslinker FPOB with dual electron‐withdrawing groups of trifluoromethyl (─CF_3_) and ether‐oxygen bonds (─O─) was synthesized through a three‐step reaction sequence, and ^1^H NMR results for products are shown in Figures S1–S3 (Supporting Information), respectively. The Gaussian 16W program was employed to optimize the geometry of copolymerized components, and no imaginary frequency is observed in **Figure** **1**a according to the vibrational frequency theory. Amphiphilic block copolymers containing different molar ratios of PEA (5% and 10%) were successfully synthesized by low‐temperature polycondensation combined with a gradient warming imidization, and the upper content of PEA was set as 10% to avoid the rising dielectric loss caused by the insufficient thermal stability. The hydrophobic FPI chain is connected to the hydrophilic PEA component via a covalent bond, forming a microphase‐separation structure due to the thermodynamic incompatibility between the block segments.^[^ ^32^ ^]^ Based on Flory‐Huggins theory, the interaction parameter χ between FPI and PEA is calculated to be 18.2 (derived from the solubility parameters δ_1_ = 23 (cal cm^−3^)^1/2^ for FPI and δ_2_ =  18 (cal cm^−3^)^1/2^ for PEA,^[^ ^33^, ^34^ ^]^ and the details calculation could be found in Supporting Information), which exceeds the critical threshold for microphase separation (χ_N_ ≥ 10.5) and confirms their strong thermodynamic incompatibility. Meanwhile, the mixing entropy Δ*S*_m_ = 2.7 J/(mol·K) is estimated for 10% PEA content, and the positive value reflects the entropic driving force for spontaneous self‐assembly. The schematic structure of the aggregation state of crosslinking cFPI‐PEA is demonstrated in Figure 1b. The introduction of PEA proportion is also confirmed by FT‐IR results in Figure S4 (Supporting Information), in which the characteristic C─H stretching vibrational peaks are observed in the range of 2800–3000 cm^−1^, e.g., νCH_2_ of 2925 cm^−1^ and νCH_3_ of 2854 cm^−1^. Meanwhile, the process of the imidization reaction with the complete acyl ring formation is verified by the characteristic absorption peaks, e.g., the bimodal structure with νC═O symmetric stretching of 1710 cm^−1^ and νC─N stretching of 1364 cm^−1^ as well as acyl ring out‐of‐plane bending vibration of 710 cm^−1^.

The PEA segments are distributed in the FPI matrix as dispersed phases, and the introduction of FPOB crosslinker effectively inhibits the thermokinetic destabilization by reaction between amine groups and anhydride groups from the FPI segment to yield a 3D network architecture. Density‐of‐state analysis based on Multiwfn software (Figure 1c,d) reveals that the lowest unoccupied molecular orbital (LUMO)‐highest occupied molecular orbital (HOMO) energy gap of the FPI segment is 3.49 eV, while the PEA phase exhibits a large bandwidth of 7.31 eV. Notably, the LUMO energy level difference is as high as 5.28 eV, which increases the energy barrier required for carriers to jump across the phase boundary (Δ*E* > 5 eV), resulting in the possible trapping and capture of the charges at the interface. Electron density of the FPOB molecule in Figure 1e indicates that the F atom (Mulliken charge of −0.29 e) and the ether oxygen atom (−0.60 e) of ─CF_3_ group induce a strongly localized electron potential. The synergistic effect of this dual electron‐absorbing group provides the crosslinking network a deep trapping potential, which contributes to the depression of conductivity loss for the received copolymer with the refined nanophase.

**Figure 2:**
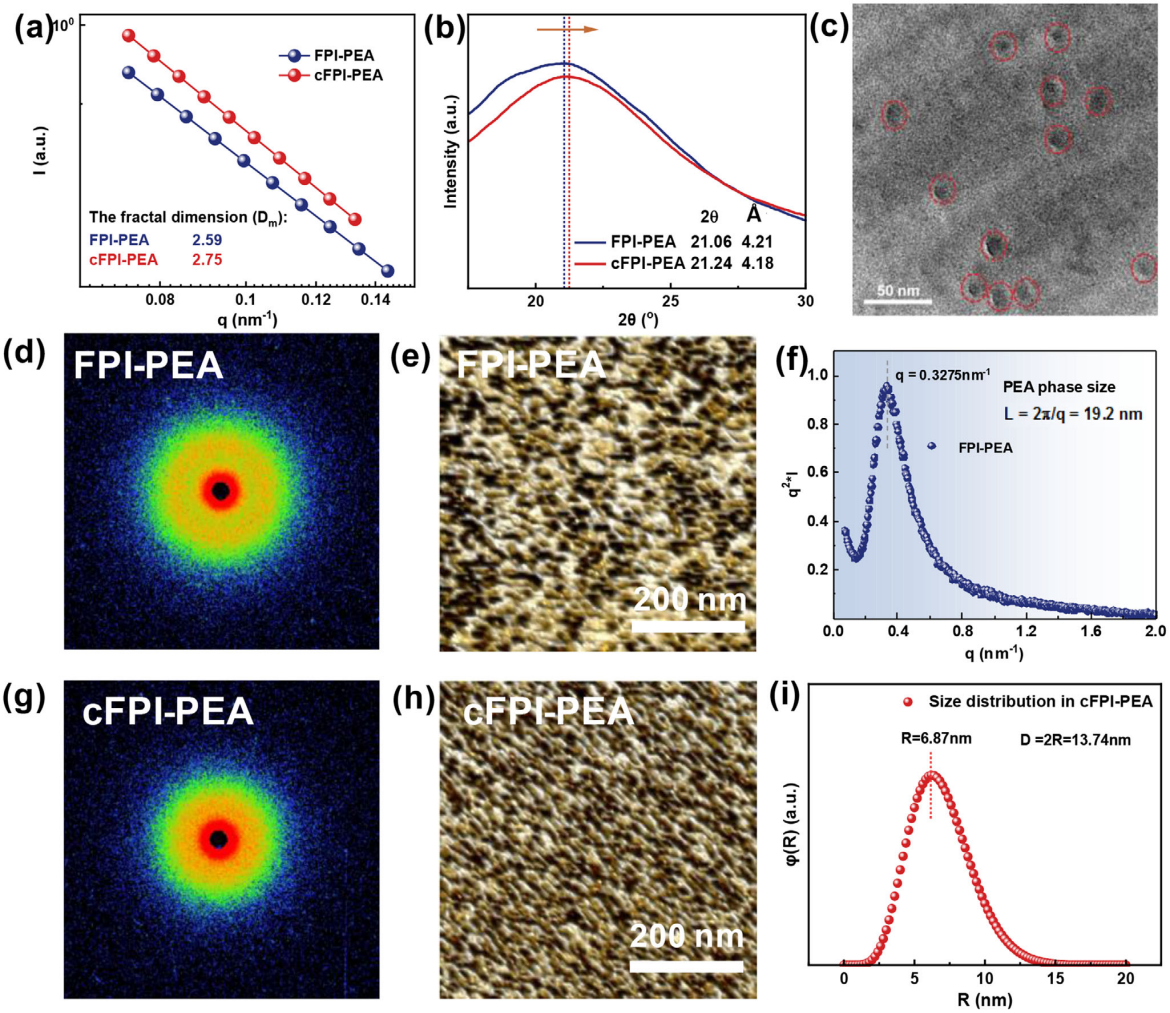
Crosslinking‐induced refinement and structural characterizations of cFPI‐PEA film: a) fractal dimension of amphiphilic block copolymer from SAXS curves, b) XRD patterns, c) TEM image; d) SAXS 2D diffractogram, e) AFM image, and f) the size distribution of PEA phase for FPI‐PEA film; g) SAXS 2D diffractogram, h) AFM image, and i) the size distribution of PEA phase for cFPI‐PEA film. The solid lines are drawn to guide the eyes.

To investigate the morphological evolution driven by thermal incompatibility of the blocking components, the fine structures of crosslinking cFPI‐PEA have been characterized systematically. The double logarithmic curves of small angle X‐ray scattering (SAXS) from **Figure** **2**a illustrate the typical fractal features, from which the fractal dimension (*D*_m_) for cFPI‐PEA with *D*_m_ = 2.75 is significantly higher than *D*_m_ = 2.59 for linear FPI‐PEA based on the linear fitting results of the low *q*‐region. From XRD patterns in Figure 2b, the molecular chain spacing shrinks from *d* = 4.21 Å for FPI‐PEA to *d* = 4.18 Å for cFPI‐PEA, suggesting that the stacking density of aggregation for the copolymer is enhanced, further supporting that a dense 3D topology is generated after the tailored refinement. In TEM images of Figure 2c and Figure S5 (Supporting Information), the dark dispersed areas (marked with red circles) correspond to the PEA phase, while the continuous matrix is the FPI phase. This assignment is also supported by supplementary AFM images in Figure S6 (Supporting Information), which clearly demonstrate the content dependence of phase separation. In accordance with the large χ value with the high proportion of PEA based on Flory‐Huggins theory, the phase separation is sufficient to yield the ideal structure of “continuous rigid skeleton‐discrete flexible phase domain”.

The SAXS 2D patterns in Figure 2d,g illustrate that the linear system in the appearance of an orange diffraction halo, while the diffraction for the crosslinking system is significantly weakened. It's proposed that the integral scattering intensity reflects the evolution of the electron density in the individual phase region during the crosslinking process:^[^ ^35^ ^]^

(1) $$\int q^{2}I\left(q\right)dq=2\pi^{2}r_{e}^{2}{\left(\Delta \rho_{e}\right)}^{2}\varphi \left(1-\varphi \right)$$

$\int q^{2}I\left(q\right)dq$ represents the scattering area, *r*_e_ the effective radius of the particles or phase domains, Δρ_e_ the difference in electron density between the phases or components of the material, 𝜑 the volume fraction of one phase, and (1−𝜑) is the volume fraction of the other phase. According to the calculation in Figure S7a (Supporting Information), the scattering integral area of the crosslinking system is decreased by 36%, indicating that the crosslinking effect suppresses the electron density, which alleviates the field distortion at the interface and thus reduces the risk of partial discharge under high field. Lorentz correction to the SAXS 1D curve in Figure 2f demonstrates a characteristic peak for the linear copolymer (q* = 0.3275 nm^−1^), which is assigned to the long period of the PEA region *L* = 2π/q* = 19.2 nm. For the crosslinking system, a spherical scattering model is utilized for fractal fitting:

(2) $$I_{total}\left(q\right)=\sum_{R_{1}}\varphi_{1}\left(R_{1}\right){F_{1}}^{2}\left(q\right)$$

(3) $$F_{1}\;\left(q\right)=\frac{3\left[\sin\left(qR\right)-qR\cos\left(qR\right)\right]}{{\left(qR\right)}^{3}}$$

where *I*_total_ is the total scattering data, *R*_1_ the size of the scatterer, *φ*_1_ the volume fraction of the scatterer, *F*_1_ the scattering shape factor of the scatterer, *R* the radius of the sphere, and *q* is the magnitude of the scattering vector. The fitting process and resultant curves are displayed in Figure S7b,c (Supporting Information). The average diameter of the PEA region of the crosslinking film in Figure 2i is decreased to 13.7 nm, which is 29% smaller than that of the linear system. The AFM phase diagrams are displayed in Figure 2e,h, and the PEA phases are dispersed homogeneously in the FPI matrix. This crosslinking‐induced PEA domain refinement arises from synergistic thermodynamic adjustment and chemical interactions. DMA curves in Figure S8 (Supporting Information) reveal the combined effects of PEA content on molecular chain mobility and mechanical stability. The resultant copolymer maintains a high modulus above 1.8 GPa below 200 °C, which is close to 1.9 GPa for pure FPI. The glass transition temperature (*T*_g_) of the copolymer decreases to 257 °C for FPI‐PEA film, which illustrates that the introduction of flexible PEA segments reduces the energy barriers of chain mobility. DMA curves in Figure S9 (Supporting Information) demonstrate that the crosslinking system retains a high modulus over a wide temperature range of 40–300 °C, e.g., 1.3 GPa for cFPI‐PEA and 0.35 GPa for FPI‐PEA at 250 °C. The temperature of peak loss shifts from 257 to 290 °C, which is attributed to the restricting effect of the network on the thermal movement of the macromolecular chains, diminishing the risk of mechanical failure under high temperature.

### 2.2 Dielectric Response and Energy Storage Capability of cFPI‐PEA Film

**Figure 3:**
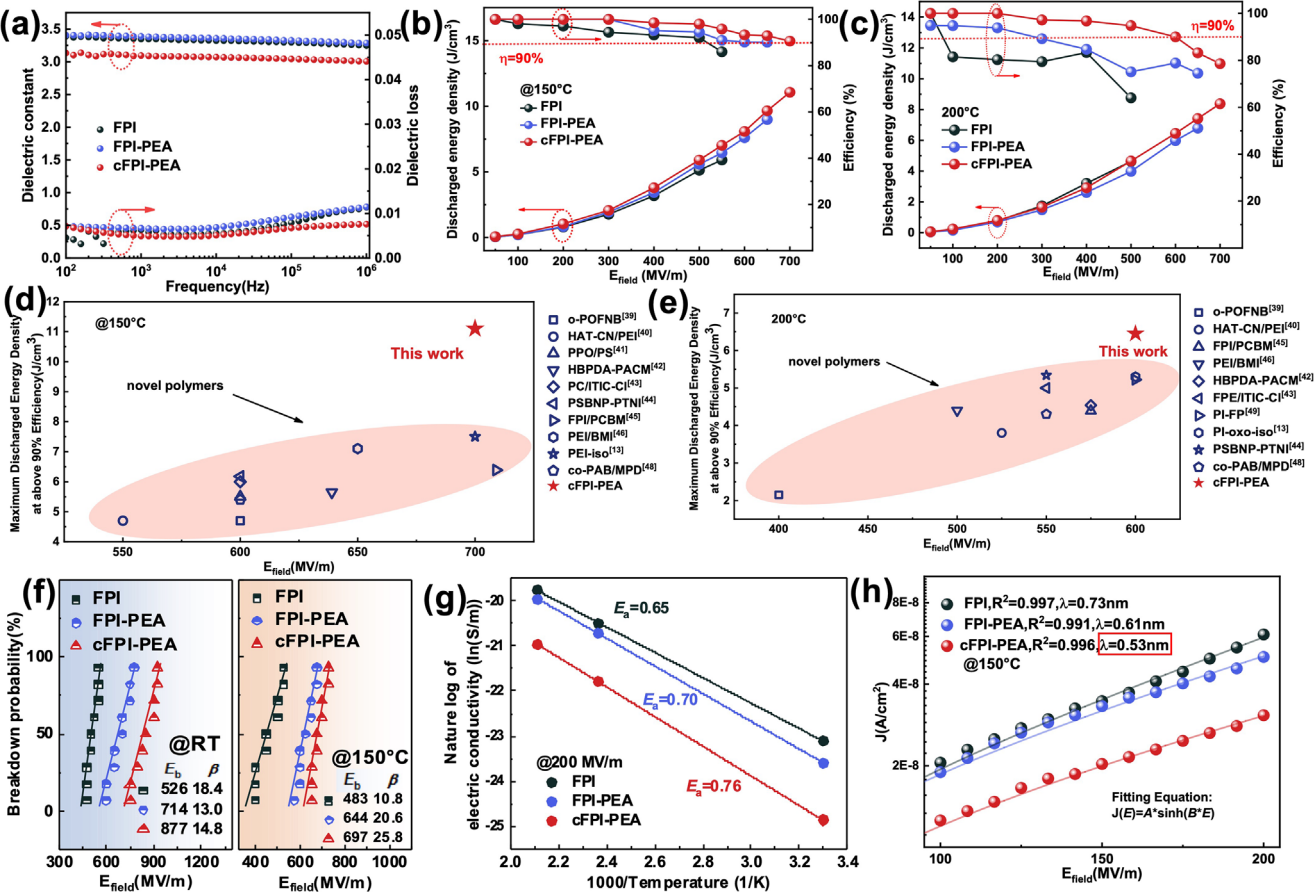
Dielectric property and energy storage capability of cFPI‐PEA film: a) dielectric constant and dielectric loss versus the testing frequency, b) energy density and charge–discharge efficiency versus the testing electric fields at 150 °C, c) energy density and charge–discharge efficiency versus the testing electric fields at 200 °C; comparison of energy density with efficiency >90% under typical all‐on‐base dielectric fields operating at 150 °C, d) and 200 °C; e,f) breakdown strengths at 25 °C and 150 °C, g) temperature‐dependent conductivity, and h) leakage currents at 150 °C with *J*–*E* conduction fitting. The solid lines are drawn to guide the eyes.

The fluoropolymer with great potential in the field of dielectric energy storage is due to the unique fluorine elemental effects, including strong electronegativity and dipole moment, as well as spatial site resistance.^[^ ^36^, ^37^, ^38^ ^]^ By introducing hydrophilic PEA segments into the FPI matrix through a molecular engineering strategy, an all‐all‐organic FPI‐PEA copolymer with amphiphilic phase‐separation aggregation has been constructed. The dielectric curves of FPI‐PEA with different PEAs are demonstrated in Figure S10 (Supporting Information). The dielectric constant of pure FPI is 3.35 at 1 kHz due to the close stacking of rigid segments. When the content of PEA increases to 10%, the contribution of oriented polarization is strengthened, caused by the high density of the ether bond, and the dielectric constant reaches 3.40 at 1kHz. Also, the frequency‐dependent tests indicate that the dielectric loss of the 10% PEA system is always lower than 0.0012 in the testing range of 100 Hz–1 MHz. In order to investigate the effect of the refined nanophase on the dielectric performance, the dielectric curves versus the testing frequency are displayed in **Figure** **3**a. The copolymer films with different concentrations of crosslinker FPOB are also prepared to explore the formation of a crosslinking network, and the dielectric property is shown in Figure S11 (Supporting Information). The energy storage performance exhibits a clear dependence on PEA content (Figures S12 and S13, Supporting Information). In a 5% PEA system, the sparse distribution of the PEA region leads to a limited inhibitory effect on carrier migration. However, under the conditions of 150  C and 600 MV m^−1^, its energy density reaches 7.53 J cm^−3^ and the efficiency is 88.6%. In a 5% PEA system, the sparse distribution of the PEA region leads to a limited inhibitory effect on carrier migration. When the PEA content increases to 10%, the phase separation structure becomes more complete. However, under the conditions of 150 °C and 600 MV m^−1^, its energy density reaches 7.53 J cm^−3^ and the efficiency is 88.6%. As can be seen from the atomic force microscopy phase images, the proportion of dispersed black PEA regions increases, forming a denser migration barrier network. This structure significantly inhibits long‐range carrier transport, as evidenced by the reduction in leakage current density from 6.10 × 10^−8^ A cm^−2^ (0% PEA) to 5.04 × 10^−8^ A cm^−2^ (10% PEA) at 200 MV m^−1^ (Figure 3h). Consequently, the 10% PEA system delivers 9 J cm^−3^ with 90.1% efficiency at 150 °C and 650 MV m^−1^, validating that PEA content optimizes domain distribution to enhance energy storage performance. Compared with the pristine linear copolymer, the dielectric constant of cFPI‐PEA slightly decreases to 3.10 at 1 kHz, which is attributed to the spatial confinement effect of the crosslinking network on the orientational polarization. Although the introduction of flexible PEA segments induces dipole relaxation by enhancing the chain orientation motion, the formation of a crosslinking network effectively suppresses the conformational relaxation of the molecular chains, which results in the cFPI‐PEA film with optimal frequency domain stability.

The unipolar loops and energy storage capability of FPI‐PEA with different PEA contents are displayed in Figures S12 and S13 (Supporting Information). The testing temperature is 150 °C. The energy storage property of cFPI‐PEA has been evaluated based on the electric hysteresis loop system over a wide temperature range of 25–200 °C. *P*‐*E* loops at 25 and 150 °C for cFPI‐PEA films with different additions of FPOB are demonstrated in Figures S14 and S15 (Supporting Information), respectively. To further examine the advantage of the amphiphilic system under a high‐temperature electric field, the hysteresis loops of FPI, FPI‐PEA, and cFPI‐PEA under a testing temperature of 200 °C are displayed in Figure S16 (Supporting Information). The energy density and charge–discharge efficiency at 150 °C versus the testing electric fields are plotted in Figure 3b, and the crosslinking film exhibits efficient energy storage performance. For example, cFPI‐PEA film achieves a maximum discharge capacity of *U*_e_ = 22.34 J cm^−3^ and an efficiency of *η* = 84.16% at 900 MV m^−1^ and 25 °C (Figure S17, Supporting Information), and *U*_e_ = 11.07 J cm^−3^ with *η >* 90% at 700 MV m^−1^ is obtained under the testing temperature of 150 °C (Figure S18, Supporting Information). The amphiphilic system has a significant advantage in maintaining remarkable efficiency, which is ascribed to the phenomenon that the introduction of PEA isolates the π–π conjugation in FPI, while its highly insulating phase prevents carrier diffusion, thus reducing the energy loss at high field. The energy storage performance at 200 °C for cFPI‐PEA is also characterized in Figure 3c, and *U*_e_ = 6.45 J cm^−3^ with *η =* 90% at 600 MV m^−1^ is achieved in the optimum film. As a representative performance metric, the energy storage capability of cFPI‐PEA at the elevated temperature exceeds that of most of the reported all‐aryl‐based polymer dielectrics,^[^ ^13^, ^39^, ^40^, ^41^, ^42^, ^43^, ^44^, ^45^, ^46^, ^47^, ^48^ ^]^ as shown in Figure 3d (the testing temperature of 150 °C) and 3e (the testing temperature of 200 °C). The crosslinking structure retains the phase stability at high temperature with the heat resistance, effectively resisting the conductivity loss with intrinsic breakdown under high‐temperature electric field. 

The Weibull statistical analysis of breakdown performance in Figure 3f validates the strengthening mechanism, and cFPI‐PEA film exhibits a characteristic breakdown strength (*E*_b_) of 877 MV m^−1^, a huge enhancement compared with the pristine FPI of *E*_b_ = 526 MV m^−1^. The temperature‐dependent conductivity in Figure 3g and Figure S19 (Supporting Information) demonstrates that cFPI‐PEA has the highest activation energy of 0.76 eV compared with 0.65 eV for FPI, confirming the significant enhancement of its carrier transport barrier. This enhancement effect stems from the synergistic effect of the crosslinking network and the amphiphilic phase separation: 1) the PEA phase domain restricts the charge migration paths; 2) the introduction of high‐polar crosslinking network increases the trap density, compressing the charge hopping distance from 0.73 to 0.53 nm based on the *J*–*E* model fitting in Figure 3h, resulting in a decrease of the leakage current density at 200 MV m^−1^ (from 6.10 × 10^−8^ A cm^−2^ for FPI to 3.07 × 10^−8^ A cm^−2^ for the resultant copolymer).

### 2.3 Cycling Stability and Anti‐Strike‐Through Mechanism of cFPI‐PEA Film

**Figure 4:**
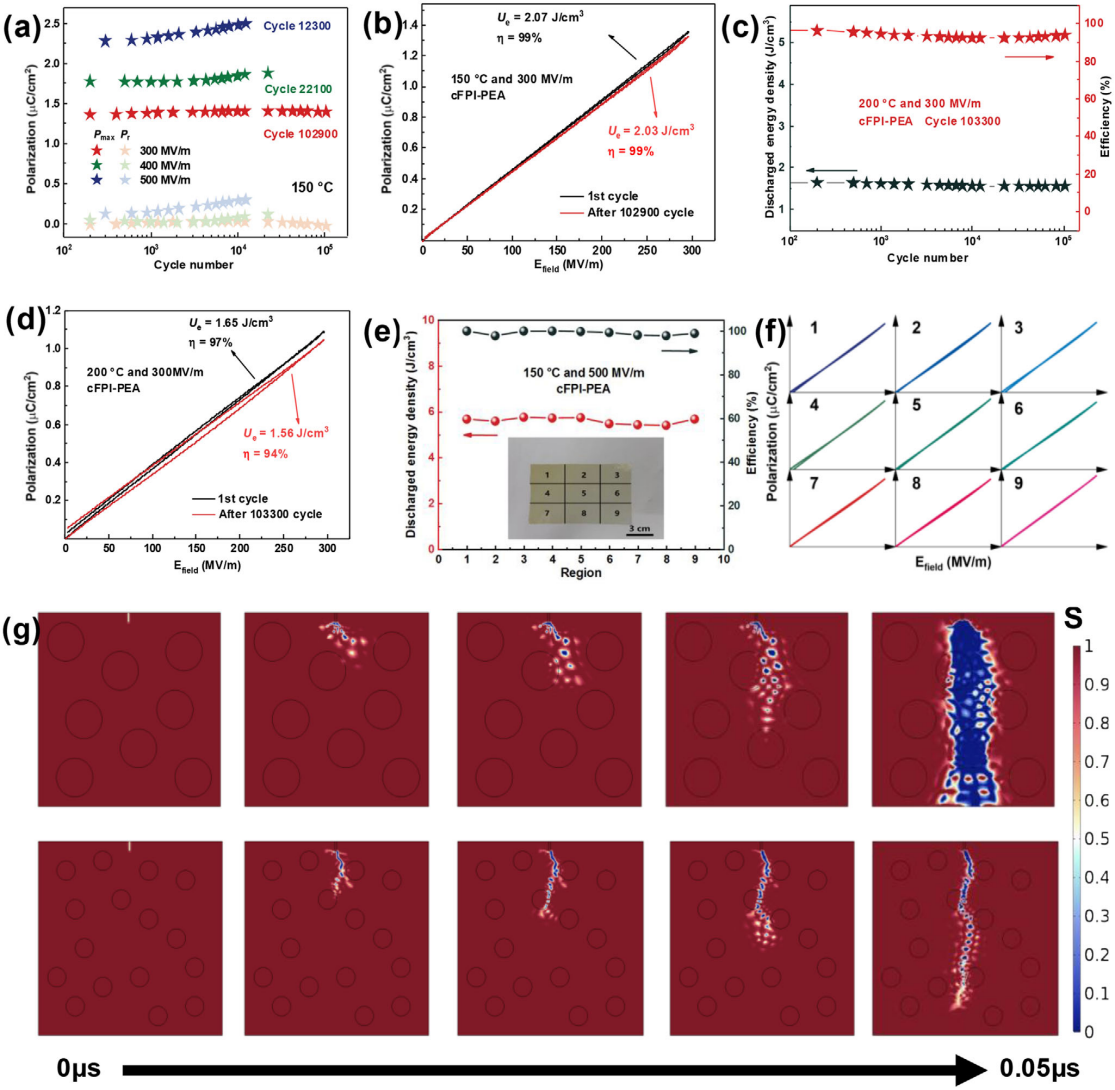
Cycling stability of cFPI‐PEA film: a) the cyclic performance under different testing fields at 150 °C, b) comparison of *P‐*‐*E* loops after 102,900 cycles at 300 MV m^−1^, c) the evolution of discharged energy density and efficiency versus testing cycles at 300 MV m^−1^ and 200 °C, d) comparison of *P*–*E* loops after 103,300 cycles at 300 MV m^−1^ and 200 °C, e) the evolution of energy density and efficiency with different regions of the received film at 500 MV m^−1^ and 150 °C, f) the corresponding *P*–*E* loops at 500 MV m^−1^ and 150 °C, and g) finite element simulation of electric dendrite growth process in copolymer film with refined size of PEA phase. The solid lines are drawn to guide the eyes.

The cFPI‐PEA system exhibits high‐temperature field strength tolerance, and its low‐loss feature extends the operating life of the film capacitor. From the electric cycling characterizations at 150 °C in **Figure** **4**a, the crosslinking film remains stable output after 102 900 charge‐release circles with an external field of 300 MV m^−1^, and the cycle number achieves 12 300 even at extreme strength of 500 MV m^−1^. The comparison of the slim *P*–*E* loop after 10^5^ cycles is displayed in Figure 4b, from which the energy density slightly decreases from 2.07 J cm^−3^ to 2.03 J cm^−3^ with *η =* ≈99% at 150 °C and 300 MV m^−1^. Under the harsh condition of 200 °C (Figure 4c,d), the energy density is 1.56 J cm^−3^ with *η =* 94% under 300 MV m^−1^ after 10^5^ cycles. In order to verify the reliability of the film preparation, the nine‐point sampling method was utilized to evaluate the regional performance under 500 MV m^−1^ and 150 °C (Figure 4e,f), and *P*–*E* loop of each region demonstrates highly overlapping elongated return curves, which evidences the film with thickness uniformity and structural consistency that could satisfy the requirements of large‐scale production.

The anti‐electrical breakdown mechanism of the phase‐separation structure is examined by constructing a phase‐field damage evolution model, defining the scalar field *s*(x,y,t)∈[0,1] to characterize the dielectric breakdown state with *s* = 1 for the intact state and *s* = 0 for the failed state.^[^ ^49^, ^50^ ^]^ The simulations in Figure 4g illustrate that the crosslinking‐induced refinement of the PEA phase region, decreasing from 19.2 to 13.7 nm, increases the zigzagging of the electrical dendritic growth paths, which results in the trapping probability of charge at the PEA phase/interface, significantly retarding the time evolution rate of the *s*‐field. The optical morphologies of copolymer films after the breakdown tests are shown in Figure S20 (Supporting Information). The images of the breakdown regions reveal distinct morphological differences between crosslinking cFPI‐PEA and linear FPI‐PEA. The crosslinking sample exhibits a small, irregular breakdown hole. This difference reflects that crosslinking‐induced PEA domain refinement affects the electrical dendritic growth paths. Meanwhile, crosslinking film has minimal charring, as the refined PEA domains form an interfacial network that restricts local decomposition caused by overheating, validating the enhanced microstructural stability. The nano‐limited domain strategy suppresses the accumulation of space charge by increasing the scattering path and trapping density, which contributes to the enhanced breakdown threshold and the reliable operation of the film capacitor.

### 2.4 Design of Highly Polar Crosslinking Agents and the Pervasive Energy Storage Application

**Figure 5:**
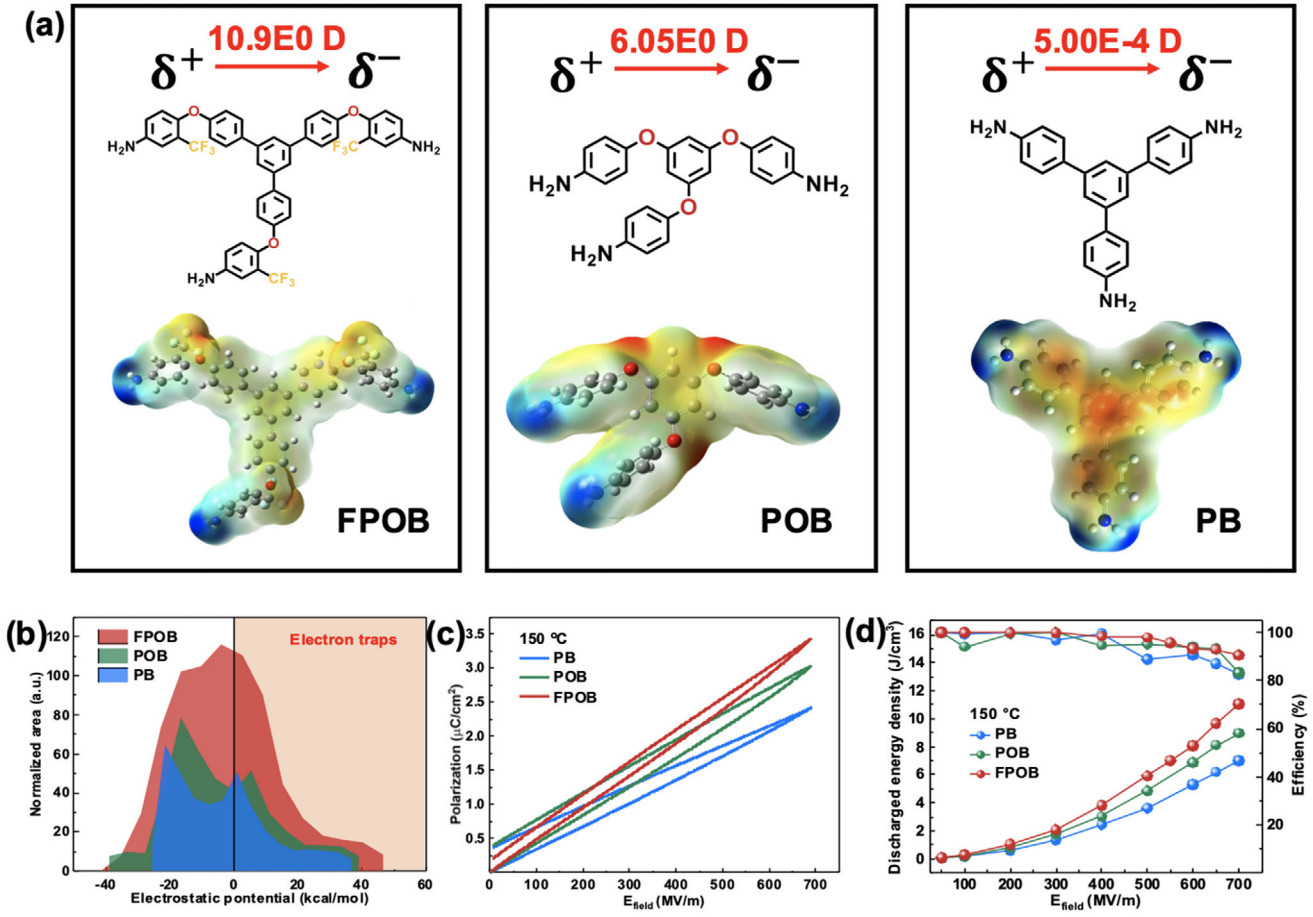
The comparison of energy capability for cFPI‐PEA films with different crosslinking agents: a) electrostatic potential distribution and dipole moment, b) area normalization of electrostatic potential, c) *P‐*‐*E* loops at 150 °C and 700 MV m^−1^, and d) energy density and efficiency versus testing electric fields at 150 °C. The solid lines are drawn to guide the eyes.

Although a conventional crosslinking network reduces the conduction loss (σ < 1 × 10^−15^ S cm^−1^) by restricting chain segment motions through covalent bonding, the low‐polarity central structure inhibits dipole orientation polarization, resulting in a limited enhancement of the potential shift under high electric field. The tailored crosslinker FPOB (**Figure** **5**a) containing trifluoromethyl (─CF_3_) and ether bonds (─O─) reaches a molecular dipole moment of 10.9 Debye calculated by B3LYP/6‐31G(d,p) theory, which is higher than that of the commercial crosslinker POB (6.05 Debye) and PB (5.00 × 10^−4^ Debye). The dielectric curves of cFPI‐PEA incorporating different crosslinkers are demonstrated in Figure S21 (Supporting Information). At 1 kHz, the dielectric constant (*ε*_r_) illustrates a positive correlation with the dipole moment of the crosslinker, e.g., *ε*_r_ = 3.10 for cFPI‐PEA with FPOB, *ε*_r_ = 3.06 for film with POB, and *ε*_r_ = 3.05 for film with PB. All these crosslinking systems maintain low dielectric loss (< 0.01) over the frequency range of 10^2^–10^6^ Hz, which is attributed to the constrained molecular motion by the crosslinking network. Quantitative analysis of the electrostatic potential surface by Multiwfn software (Figure 5b) demonstrates that the area share of FPOB in the positive potential energy region is much higher than POB and PB, which is attributed to the synergistic effect of the strong electron‐absorbing effect of ─CF_3_ with lone‐pair electrons of ether bonding, resulting in the deep electron traps for carrier migration,^[^ ^40^, ^43^ ^]^ which is consistent with its highest Weibull breakdown strength at 150 °C (Figure S22, Supporting Information). Three types of crosslinkers have been introduced into FPI‐PEA systems to investigate the evolution of electric displacement and energy storage capability (Figure 5c,d; Figure S23, Supporting Information). The polarization at 700 MV m^−1^ for FPOB crosslinking system reaches 3.43 µC cm^−2^, which is much higher than that of POB (3.03 µC cm^−2^) and PB (2.41 µC cm^−2^). It's noteworthy that the microphase‐separation systems with commercial crosslinkers still maintain outstanding high‐temperature energy storage performance, e.g. at 150 °C, *U*_e_ = 8.10 J cm^−3^ with  *η* = 90% for the POB system and *U*_e_ = 3.61 J cm^−3^ with  *η* = 90% for the PB system, evidencing that the refinement of entropy‐driven microphase separation in dielectric copolymer is an effective method to improve the energy storage capability of polymer film.

## 3 Conclusion

In summary, an amphiphilic dielectric copolymer is developed through innovative molecular design and nanophase engineering. Through the introduction of crosslinking molecules with strong electron‐absorbing groups for synergistic regulation, the “rigid‐flexible” nature of the material structure is realized, in which the rigid chain segments construct a highly heat‐resistant skeleton, the high phase barrier in the flexible phase domain inhibits carrier migration, and the unique electron‐absorbing crosslinking sites form intrinsic charge traps to depress the conductivity loss. The resultant cFPI‐PEA film reaches *η* >90% with *U*_e_ = 11.07 J cm^−3^ at 150 °C and 700 MV m^−1^ due to the decreased hopping distance of charge carriers. The breakdown process is slowing down through the nanophase domain with fatigue resistance, and the tailored copolymer film retains *η* = 94% with *U*_e_ = 1.56 J cm^−3^ after 10^5^ charge–discharge cycles of 300 MV m^−1^ at 200 °C. This provides an innovative solution for the long‐lasting and stable operation of the next‐generation high‐power‐density film capacitor.

## 4 Experimental Section

### Materials

Reagents of silicon tetrachloride, 4‐hydroxyacetophenone, palladium on carbon, 4,4′‐diamino‐2,2′‐bis(trifluoromethyl)biphenyl (TFMB), and 2‐chloro‐5‐nitro‐trifluoromethyltoluene were purchased from Shanghai Aladdin Biochemical Technology Co. (2‐Aminopropyl)poly(propylene glycol)‐block‐poly(ethylene glycol)‐block‐poly(propylene glycol) (PEA, *M*_n_ = 500 g mol^−1^) was obtained from Macklin Biochemical Co. N,N‐dimethylacetamide, ethanol, hydrazine hydrate, potassium carbonate (K_2_CO_3_), N‐methylpyrrolidone (NMP), and 3,3',4,4′‐biphenyltetracarboxylic dianhydride (BPDA) were supplied by Sinopharm Chemical Reagent Co. All chemicals were used as received without further purification.

### Synthesis of 1,3,5‐tris(4‐(2‐trifluoromethyl‐4‐aminophenoxy)phenyl)benzene

First, under the protection of nitrogen, 0.1 mmol of 4‐hydroxyacetophenone was refluxed with 0.125 mmol of K_2_CO_3_ in 80 mL of DMAC at 120 °C for 3h, and the reaction was continued with the addition of 0.1 mmol of 2‐chloro‐5‐nitro‐trifluoromethyltoluene for 12 h. The product of 1,4‐(2‐trifluoromethyl‐5‐nitrophenoxy)acetophenone was obtained by alcoholic precipitation and recrystallization in alcohol/water. Then, it was reacted with 14 mL of silicon tetrachloride in 40 mL of ethanol for 14 h, and the mixture was purified by ethanol reflux and recrystallized from mixed solvents, and 1,3,5‐tris(4‐(2‐trifluoromethyl‐4‐nitrophenoxy)phenyl)benzene was received. Finally, the above sample was refluxed with 0.6 g of palladium carbon in ethanol at 80 °C, and reduced by 20 mL of hydrazine hydrate for 12 h. After precipitation by water, the light‐yellow powder 1,3,5‐tris(4‐(2‐trifluoromethyl‐4‐aminophenoxy)phenyl)benzene (FPOB) was obtained. The whole process involves nucleophilic substitution, benzene cyclization, and catalytic reduction reaction, and the key steps include multiple recrystallization purification and hydrolysis post‐treatment.

### Synthesis of Fluorinated Polyamido Acid

In a three‐neck flask equipped with a magnetic stirrer, 3 mmol of TFMB was added and dissolved in 4.34 mL of NMP under a nitrogen atmosphere. The reaction system was maintained at 0 °C using an ice water bath. Additionally, 3.06 mmol of BPDA was dissolved in 4 mL of NMP and divided into three equal aliquots. The BPDA solution was then gradually added to the diamine solution every 30 min. After complete addition, the reaction mixture was stirred continuously for 24 h to ensure complete reaction.

### Synthesis of Fluorinated Amphiphilic Block Copolymer Polyamido Acids

The synthesis was carried out under nitrogen protection using a similar procedure as in the previous section, with modifications in the monomer ratios. Taking FPI‐PEA 10% as an example, a mixture containing 2.7 mmol TFMB and 0.3 mmol PEA was dissolved in 4.6 mL of NMP, and the mixture was cooled to 0 °C. Subsequently, 3.06 mmol BPMB dissolved in 4 mL of NMP was added in three additions at 30‐min intervals. The resulting viscous solution was stirred for 24 h at 0 °C to ensure complete reaction.

### Synthesis of Crosslinking Fluorinated Amphiphilic Block Copolymers Polyamidoacetic Acid

A series of crosslinking cFPI‐PEA materials was constructed by adding 0.01, 0.05, and 0.1 mmol FPOB crosslinker. The crosslinker derivatives were prepared by a sequential polymerization strategy. Taking cFPI‐PEA 0.1 as an example, 2.7 mmol of TFMB and 0.3 mmol of PEA were first dissolved in 4.6 mL of NMP under nitrogen atmosphere. After cooling the solution to 0 °C, 3.06 mmol of BPDA dissolved in 4 mL of NMP was added by a three‐step addition method as described previously. After 12 h of polymerization, 0.1 mmol of FPOB was added as a crosslinker. The reaction was continued for 12 h to incorporate crosslinker molecules into the copolymer backbone.

### Preparation of Dielectric Films

Polyamido acid solution (1.5 mL) and NMP (8.5 mL) were stirred for 2 h, and the solution was coated on a glass substrate. After gradient imidization (100 °C/1 h, 200 °C/1 h, and 300 °C/1 h), the free‐standing films were peeled off to obtain a uniform thickness of ≈8 µm for FPI, FPI‐PEA, and cFPI‐PEA.

### Characterization

^1^H NMR spectra were recorded on a 500 MHz AVANCE III spectrometer (Bruker, Switzerland) to examine the structure of FPOB with DMSO‐d6 as the solvent. Fourier Transform Infrared Spectroscopy (FT‐IR) was used to characterize the formation of the polyimide ring and the introduction of PEA chain segments. Shimadzu X'Pert PRO X‐ray diffractometer (Cu Kα radiation, *λ* = 1.5406 Å) was used to evaluate the molecular chain spacing and stacking density. Small‐angle X‐ray scattering (SAXS) measurements were performed using a Xenocs Xeuss 2.0 system equipped with a sealed microfocus X‐ray source, and the instrument was operated with Cu Kα radiation (*λ* = 1.54189 Å) at 30 W. A hybrid pixel detector (Pilatus 3R 300K, Dectris Ltd.) with 172 µm pixel size was positioned at an appropriate sample‐to‐detector distance to achieve the required *q*‐range, and scattering patterns were collected under vacuum conditions to minimize air scattering and absorption effects. The morphologies of microphase separation were examined by Bruker Dimension Icon AFM and FEI Tecnai G2 F30 TEM, and the sample film was ultrathin cut before the TEM testing. TA Instruments Q800 Dynamic Mechanical Analyzer (tensile mode with frequency of 1 Hz) was employed to evaluate the effect of crosslinking networks on thermal stability. The frequency dependence of the dielectric property was recorded by Agilent E49990A (100 Hz‐1 MHz). Unipolar polarization–electric field hysteresis (*P‐*‐*E*) loops, current–voltage (*I‐*‐*V*) curves, and leakage conductive values were acquired using a Radiant Multiferroic II ferroelectric test system at 100 Hz triangular waves. A thin gold layer of 0.0707 cm^2^ was sprayed on both sides prior to characterization. To examine the electric displacement under the elevated temperature, the test samples were immersed in dimethylsiloxane, and the controlled temperature was fed back through a thermocouple. The optical morphology of electric breakdown was observed using a BX53M‐P hot‐stage polarizing microscope (Olympus, Japan) to characterize the damage characteristics of the films after breakdown.

### Density Functional Theory Calculation

The geometrical optimization of all molecular model ground states was performed on the basis of the Gaussian 16W program with the B3LYP/6‐31G(d,p) basis set and functionals. The quantitative analysis of the surface electrostatic potentials was carried out in Multiwfn (V3.7) with the lattice spacing set to 0.25 bohr and the electron density set to 0.001 e bohr^−3^.^[^ ^51^ ^]^

### Finite Element Simulation of Electric Dendrite Growth

COMSOL Multiphysics software was utilized to simulate the FPI‐PEA and cFPI‐PEA electric dendrite growth paths, in which a scalar phase field *s*(x, y, t) is used to describe the breakdown state of the polymer dielectric, with *s* varying between 0 and 1, where *s* = 1 for the pristine state and *s* = 0 for the fully damaged state. Since a fully damaged material loses its insulating ability and becomes conductive, for simplicity, the behavior of the conductive phase is modeled using a dielectric phase with a very high dielectric constant. The electrical work done by the electric field through the conduction current is replaced by electrostatic energy released through a large potential shift. Mathematically, the dielectric constant is described as a function of the phase field variable:

(4) $$\varepsilon \left(s\right)=\frac{\varepsilon^{0}}{f\left(s\right)+\eta }$$

Here, *ε* ^0^ is the initial dielectric constant of the material, *f*(*s*) = 4*s* ^3^–3*s* ^4^ the interpolating function between *f*(0) = 0 and *f*(1) = 1, and *η* is a number added for numerical calculation with the setting of *η* = 10^−3^ corresponding to a dielectric constant for the damaged phase that's 1,000 times higher than the intact phase.

Breakdown within a dielectric material occurs when the propagation of the electric tree results in a decrease in the total potential energy of the phase field model. The total potential energy of the system consists of establishing multiple energy generalizations, including the electric field storage energy (*W_es_* (*E*,*s*)), the damage energy (*W_d_* (*s*)) and the interface energy (*W_i_* (∇*s*)) at the sharp phase boundary

(5) $$\Pi \;\left[s,\emptyset \right]=\int_{\Omega }\left[W_{es}\left(E,s\right)+W_{d}\left(s\right)+W_{i}\left(\nabla s\right)\right]dV$$

Linear dynamics was used to describe the damage evolution, and the equations of motion were established by the damage evolution equations and energy floods:

(6) $$\bar{\nabla }\cdot \left[\frac{1}{f\left(s\right)+\eta }{\bar{\nabla }}_{\emptyset }\right]=0$$

(7) $$\frac{\partial s}{\partial t}=-\frac{f^{\prime }\left(s\right)}{2{\left[f\left(s\right)+\eta \right]}^{2}}{\bar{\nabla }}_{\emptyset }\cdot {\bar{\nabla }}_{\emptyset }+f^{\prime }\left(s\right)+\frac{1}{2}\bar{\nabla }\bar{\nabla }s$$

## Conflict of Interest

The authors declare no conflict of interest.

## Author Contributions

F.Z. devised the original concept and prepared the materials, and performed the characterization. F.Z., H.Y., and L.X. analyzed the data. F.Z. and H.Y. wrote the first draft of the manuscript, and all authors participated in manuscript revision.

## Supporting information



Supporting Information

## Data Availability

The data that support the findings of this study are available in the supplementary material of this article.
