# Supplementary material for: Chain Topology Engineering in Amphiphilic Block Copolymers: Crosslinking‐Induced Nanodomain Refinement for Ultrahigh Energy Density under Extreme Conditions
Source: Adv Sci (Weinh). 2025 Sep 15;12(42):e10046. doi: 10.1002/advs.202510046 (PMC12622496; doi:10.1002/advs.202510046)
Supplement: Supplementary file 1 — Supporting Information [file ADVS-12-e10046-s001.docx]

Supporting Information

**Chain Topology Engineering in Amphiphilic Block Copolymers: Crosslinking-Induced Nanodomain Refinement for Ultrahigh Energy Density under Extreme Conditions**

*Fuxing Zhai,^a^ Lixin Xu*,^a^ Huijian Ye*^a^*

^a^ College of Materials Science and Engineering, Zhejiang University of Technology, Hangzhou 310014, China

**E-mail:** [huy19@zjut.edu.cn](mailto:huy19@zjut.edu.cn) (H. Ye), [gcsxlx@zjut.edu.cn](mailto:gcsxlx@zjut.edu.cn) (L. Xu)

**Figure S1**. ^1^H NMR result for 1,4-(2-trifluoromethyl-5-nitrophenoxy)acetophenone.

**Figure S2**. ^1^H NMR result for 1,3,5-tris(4-(2-trifluoromethyl-4-nitrophenoxy)phenyl)benzene.

**Figure S3**. ^1^H NMR result for 1,3,5-tris(4-(2-trifluoromethyl-4-aminophenoxy)phenyl)benzene.

**The mixing entropy ΔSm and interaction parameter χ**

For the interaction parameter, the repeat unit molar volumes of FPI (388.9 cm^3^ mol^−1^) and PEA (473.5 cm^3^ mol^−1^) are derived from their molecular weights and densities noted in the documents, yielding an average molar volume *v*_0_ of 431.2 cm^3^ mol^−1^. For fluorine-containing polyimide, the solubility parameter ($\delta$) is about $\delta_{1}$= 23 (cal cm^−3^)^1/2^. ^[1]^ Similarly, PEA molecular chain mainly consists of polyethylene glycol with $\delta_{2}=$18 (cal cm^−3^)^1/2^. ^[2]^ Combining the conditions of *R* = 1.987 cal/(K·mol) and *T* = 298 K, the calculated value of χ is 18.2 according to the following equation (1):

$\chi=\frac{v_{0}}{RT}\left( \delta_{1}-\delta_{2} \right)^{2}$ (1)

The interaction parameter χ =18.2 for cPFI-PEA exceeds the critical threshold for microphase separation of χ_N_ ≥ 10.5. ^[3]^ Also, the phase separation is verified by SAXS and morphological results from TEM and AFM characterizations.

The mixing entropy (Δ*S*_m_) is estimated based on the molar ratios of PEA as 5% and 10% in the synthesized copolymer. The mixing entropy Δ*S*_m_ of the block copolymer is calculated by the mixing entropy formula of Flory-Huggins theory (2):

$\Delta S_{m}=-R\left( n_{1}\ln\Phi_{1}+n_{2}\ln\Phi_{2} \right)$ (2)

where *R* is the gas constant of 8.314J/(mol·K); $n_{1}$ and $n_{2}$ are the molarities of the block segments; $\Phi_{1}$ and $\Phi_{2}$ are the volume fractions of the two embedded segments utilized to estimate the molar fraction (*e.g.* $\Phi_{\mathrm{FPI}}$= 0.9 and $\Phi_{\mathrm{PEA}}$=0.1 for 10% PEA). After calculation based on the Equ. (2), Δ*S*_m_ = 2.7 J/(mol·K) is evaluated for 10% PEA, and Δ*S*_m_ = 1.5 J/(mol·K) for 5% PEA is obtained. This positive entropy change reflects the driven force of self-assembly process, in which covalently FPI and PEA segments form nanoscale domains to balance entropy increase (disorder maximization) and energy minimization because of thermodynamic incompatibility, which is consistent with the spontaneous nanoscale microphase separation.

[1] L. S. Soh, S. U. Hong, C. Z. Liang, W. F. Yong, “Green solvent-synthesized polyimide membranes for gas separation: Coupling Hansen solubility parameters and synthesis optimization,” *Chem. Eng. J.* **2023**, *478*, 147451.

[2] K. Adamska, A. Voelkel, “Hansen solubility parameters for polyethylene glycols by inverse gas chromatography,” *J. Chromatogr. A* **2006**, *1132*, 260.

[3] K. Lee, N. Corrigan, C. Boyer, “Polymerization induced microphase separation for the fabrication of nanostructured materials,” *Angew. Chem. Int. Ed.* **2023**, *62*, e202307329.

[4] X. A. Hou, S. Chen, J. J. Koh, J. H. Kong, Y. W. Zhang, J. C. C. Yeo, H. M. Chen, C. B. He, “Entropy-driven ultratough blends from brittle polymers”, *ACS Macro Lett.* **2021**, *10*, 406.

**Figure S4**. FT-IR spectra of FPI-PEA films with different molar ratios of PEA: (a) FT-IR curves and (b) variation of methyl and methylene in the range of 2800 cm^−1^ ~ 3000 cm^−1^.

**Figure S5**. TEM images of cFPI-PEA after ultrathin sectioning.


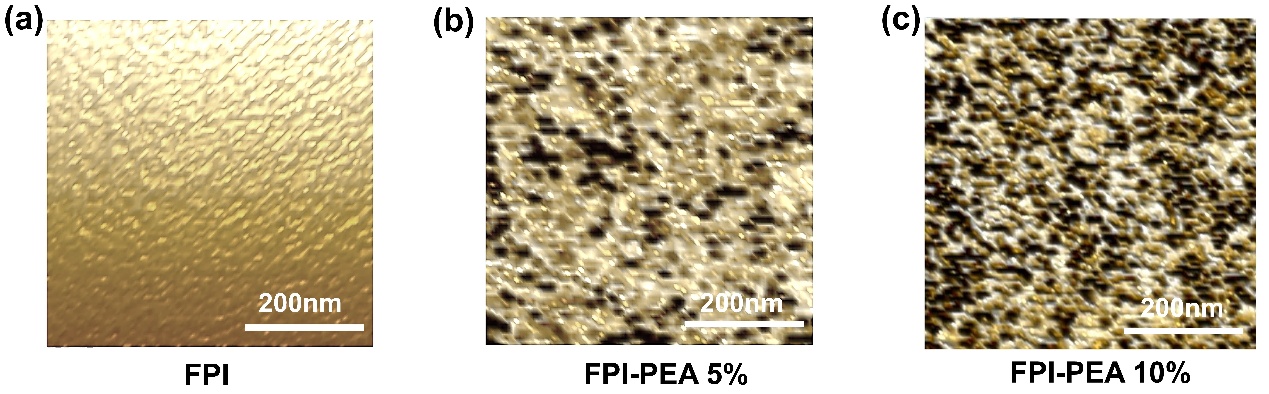


**Figure S6**. AFM phase diagrams for FPI-PEA copolymers with different PEA contents: (a) 0%, (b) 5%, and (c) 10%.


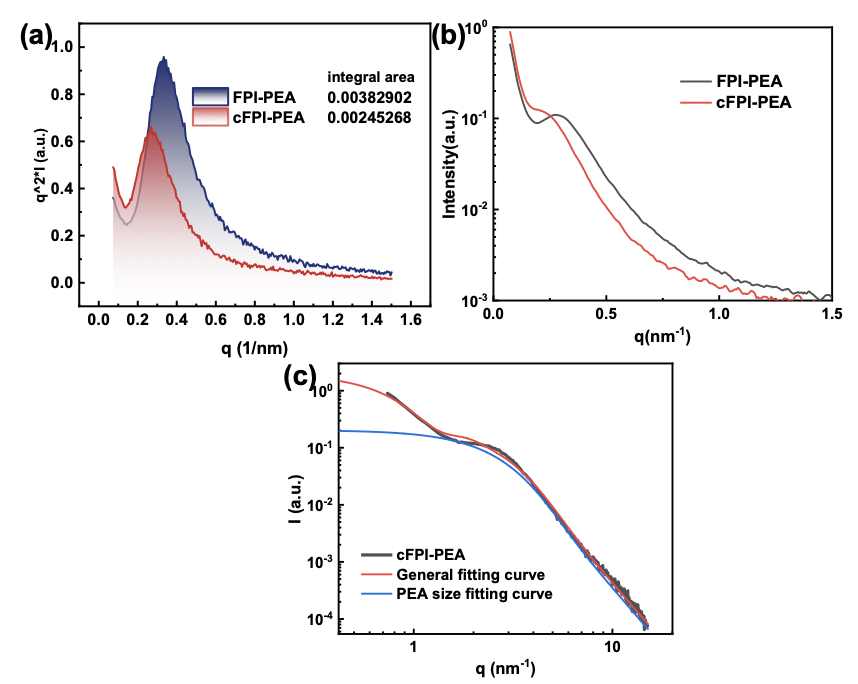


**Figure S7**. The calculation of SXAS results for FPI-PEA and cFPI-PEA: (a) Iq²-q integrals, (b) raw SAXS 1D plots, and (c) spherical scattering model fitting process.


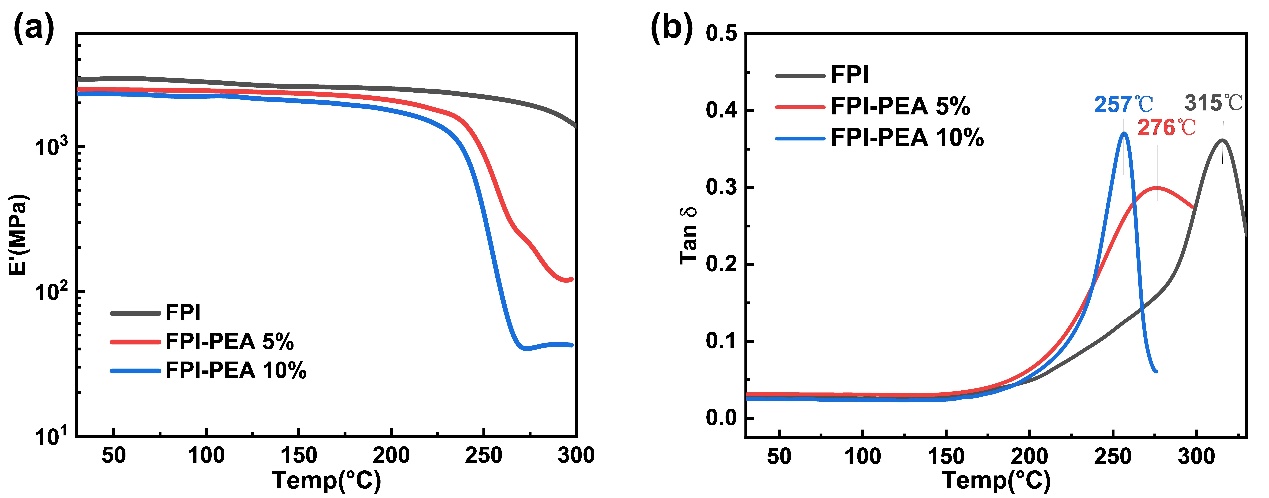


**Figure S8**. DMA results for FPI-PEA films with different PEA contents: (a) 0%, (b) 5%, and (c) 10%.

**Figure S9**. DMA curves of cFPI-PEA film: (a) energy storage modulus and (b) loss factor.


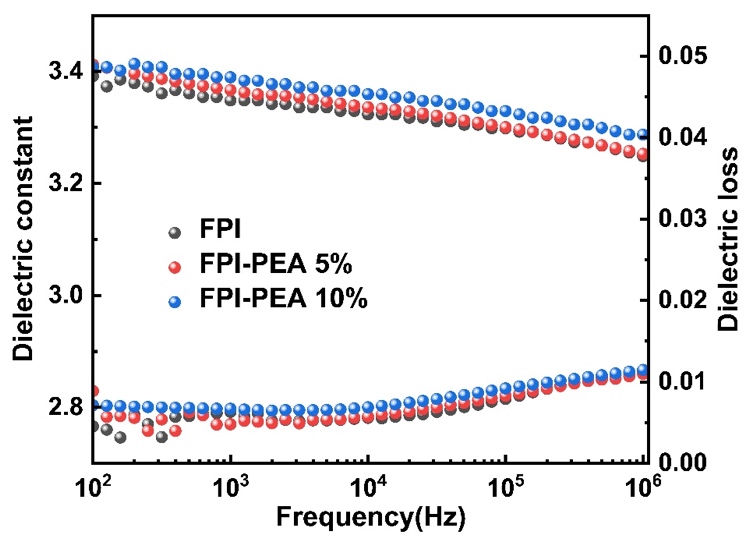


**Figure S10**. Variation of dielectric constant and dielectric loss with testing frequency for FPI-PEA with different contents of PEA.

**Figure S11**. Variation of dielectric constant and dielectric loss versus the testing frequency for cFPI-PEA with different additions of FPOB.


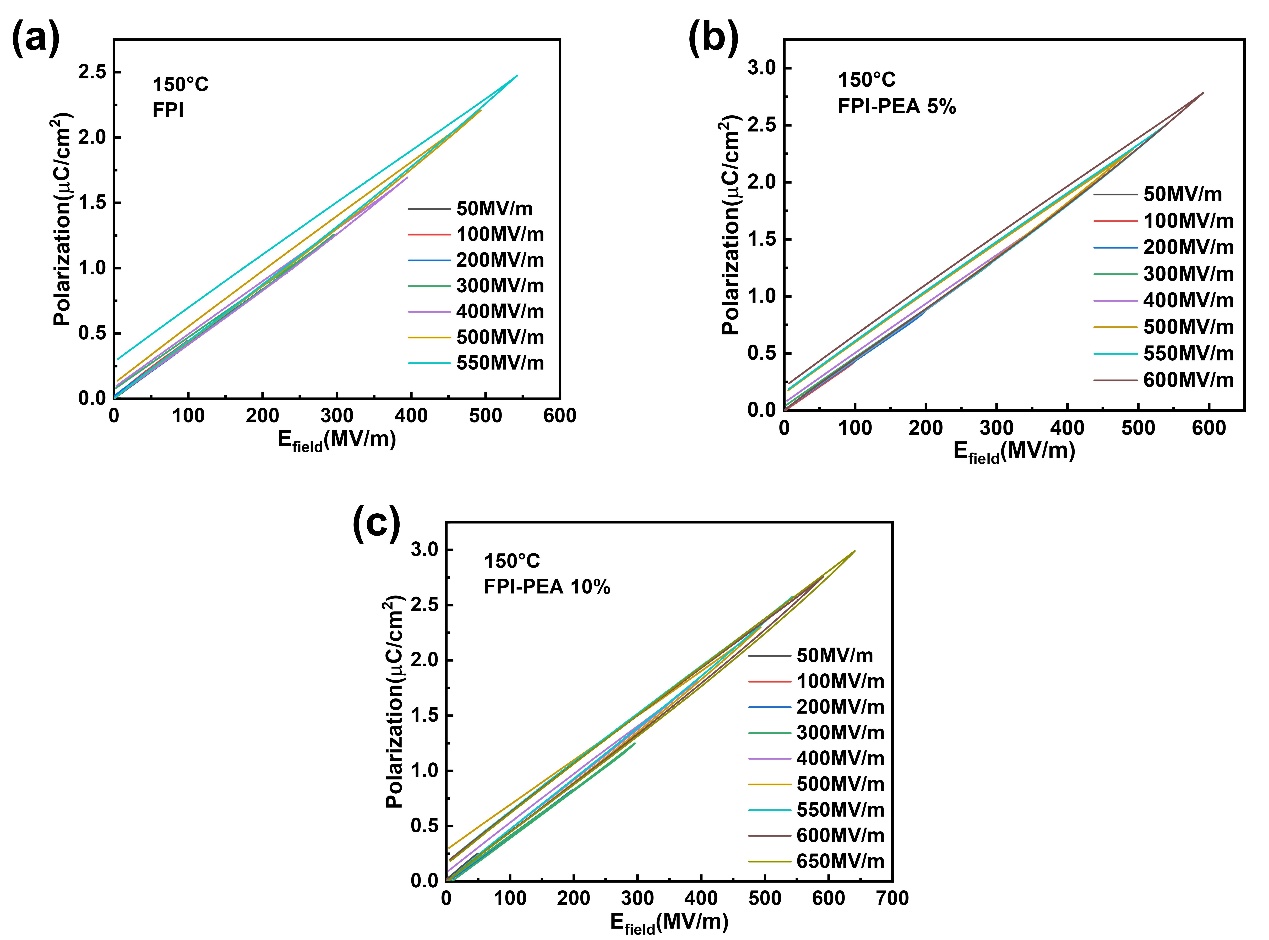


**Figure S12.** *P*-*E* loops at 150°C: (a) FPI, (b) FPI-PEA 5%, and (c) FPI-PEA 10%.


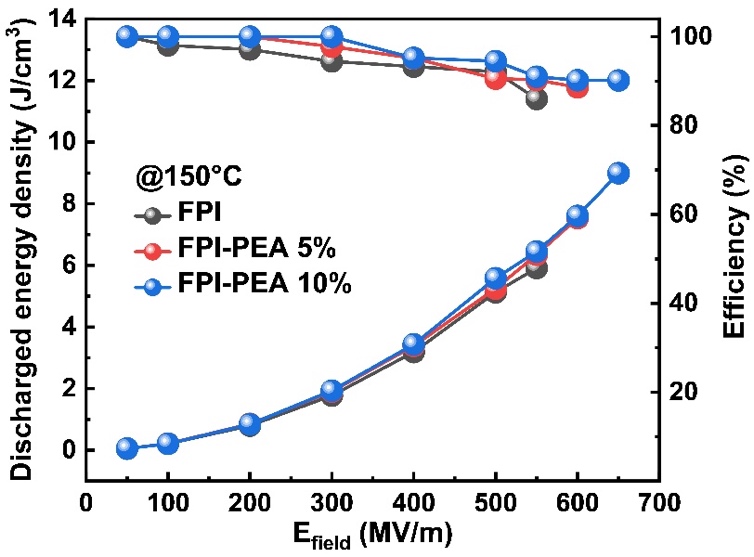


**Figure S13**. The relationship between the energy storage density and efficiency of FPI-PEA with different PEA contents and the electric field strength was investigated at 150 °C.

**Figure S14**. *P*-*E* loops under room temperature for cFPI-PEA with different additions of FPOB: (a) 0.01 mmol, (b) 0.05 mmol, and (c) 0.1 mmol.

**Figure S15**. *P*-*E* loops at 150 °C for cFPI-PEA with different additions of FPOB: (a) 0.01 mmol, (b) 0.05 mmol, and (c) 0.1 mmol.

**Figure S16**. *P*-*E* loops at 200°C: (a) FPI, (b) FPI-PEA, and (c) cFPI-PEA.

**Figure S17**. Energy storage density and efficiency versus the testing frequency at room temperature for cFPI-PEA with different additions of FPOB.

**Figure S18**. Energy storage density and efficiency versus the testing frequency at 150 °C for cFPI-PEA with different additions of FPOB.

**Figure S19**. Electric conductivity with the external field of 200 MV m^−1^ for FPI, FPI-PEA, and cFPI-PEA under different testing temperatures.


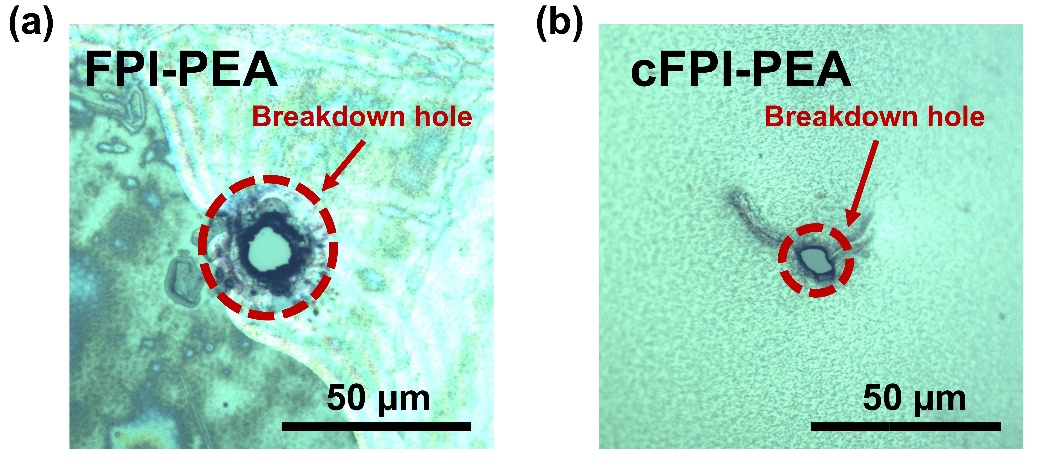


**Figure S20**. Optical photographs of thin films after electrical breakdown: (a)FPI-PEA and (b) cFPI-PEA.


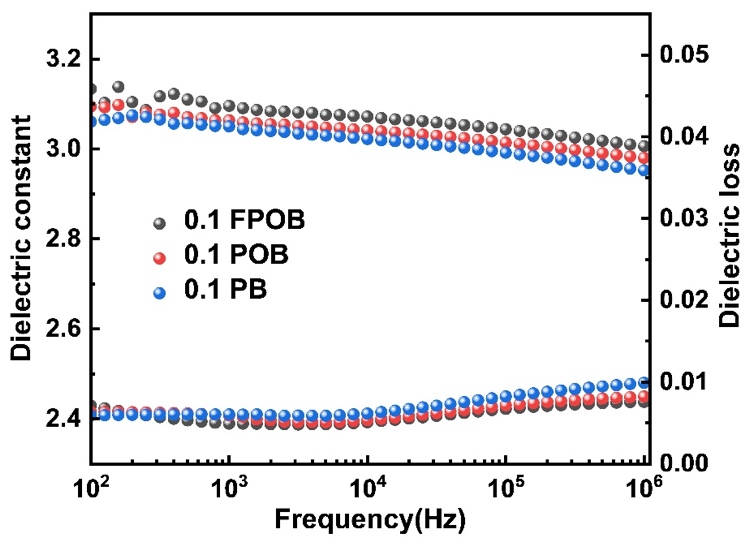


**Figure S21**. Dielectric constant and dielectric loss versus testing frequency for cFPI-PEA films with 0.1 mmol PB, POB, and FPOB.

**Figure S22**. Breakdown strength distributions of cFPI-PEA films with the addition of 0.1 mmol PB, POB, and FPOB.

Figure S23. *P*-*E* loops at 150 °C for cFPI-PEA films with 0.1 mmol PB and POB.
